# Influence of psychological intervention on pain and immune functions of patients receiving lung cancer surgery

**DOI:** 10.12669/pjms.321.8935

**Published:** 2016

**Authors:** Xinying Zhao, Limin Cui, Wei Wang, Quanzhi Su, Xiuzhi Li, Junben Wu

**Affiliations:** 1 Xinying Zhao, Binzhou People’s Hospital, Shandong 256610, China; 2 Limin Cui, Binzhou People’s Hospital, Shandong 256610, China; 3 Wei Wang, Binzhou People’s Hospital, Shandong 256610, China; 4 Quanzhi Su, Binzhou People’s Hospital, Shandong 256610, China; 5 Xiuzhi Li, Binzhou People’s Hospital, Shandong 256610, China; 6 Junben Wu, Binzhou People’s Hospital, Shandong 256610, China

**Keywords:** Psychological intervention, Lung cancer, Visual analog scale, Immune function

## Abstract

**Objective:**

To observe the influence of psychological intervention on pain, immune system and adrenocortical functions of patients receiving lung cancer surgery.

**Methods:**

We selected 124 patients who received surgery for treating stage I or II lung cancer and divided into experimental group and control group. The experimental group received comprehensive psychological intervention while the control group was given conventional nursing intervention. Pain of patients in two groups was evaluated by visual analog scale (VAS). Before and after intervention, CD3^+^, CD4^+^, CD8^+^, CD4^+^/CD8^+^ and free cortisol level in serum were measured. Moreover, QLQ-C30, a life quality measurement scale developed by European Organization for Research and Treatment of Cancer (EORTC) was used.

**Results:**

Compared to control group, VAS of patients in experimental group remarkably decreased before anesthesia, 6 hour, 12 hour 24 hour and 48 hour after surgery (P<0.05), and moreover, OLQ-C30 score and various factor scores (except physical symptoms) in experimental group were much higher (P<0.05). No statistical significant difference was found in immune index between two groups before intervention (P>0.05). Differences of CD3^+^ and CD4^+^ before and after intervention were both statistically significant (P<0.05), so did free cortisol level (P<0.05).

**Conclusion:**

Comprehensive psychological intervention can effectively relieve pain, improve immune functions and enhance quality of life for patients suffering from lung cancer surgery.

## INTRODUCTION

Surgery is the preferred treatment method for lung cancer; however, fear of surgery, incomprehension of primary disease and concern about postoperative complications result in severe depression and anxiety.^1^,^2^ Though the influence mechanism of psychological factors on prognosis of patients with cancer has not been known clearly, it has been proved that,^3^,^4^ stress stimulation caused by abnormal emotion can weaken immune functions through neuroimmunoendocrine network; changes of immune system and endocrine system severely influence recovery of patients who developed cancer; and weakened immune functions often leads to change or deterioration of cancer. In addition, most lung cancer patients have intensive pain or cough after receiving surgery.^5^ A study found that, postoperative pain is correlated to psychological situation before surgery.^6^ Therefore, it is quite important to pay attention to psychological issue during treatment of lung cancer.

At present, most researches about the correlation of psychological intervention of cancer patients with immune system and adrenocortical functions focus on chemoradiotherapy period after surgery rather than perioperative period. Additionally, correlation between postoperative pain and psychological condition before surgery is also rarely studied.

The purpose of this study was to see the influence of psychological intervention on immune function and postoperative pain of lung cancer patients in perioperative period, thus to provide a basis for psychological nursing of them.

## METHODS

### Research objects

In this study, 124 lung cancer patients who were hospitalized in cardiothoracic surgery department of our hospital between November 2010 and November 2011 were selected as research objects. Inclusion criteria included: (1) patients diagnosed with stage I or II lung cancer pathologically; (2) patients aged from 18 ~ 70 years; (3) patients who were hospitalized for the first time and have never developed diseases involving immune system and endocrine system, schizophrenia and alcohol or drug dependence; (4) patients have not received surgery, chemotherapy, radiotherapy or used immune enhancer or inhibitor; (5) patients who were willing to join the study and singed informed consent; (6) patients who had junior high school culture degree or higher. Patients were divided into experimental group and control group with random sampling method, 62 in each group. In experimental group, 46 were males and 16 were females, with mean age of (52.63± 10.65) years. As regards education background in experimental group, 37 patients (59.68%) had junior high school degree, 14 patients (22.58%) had senior high school degree, and the remaining 11 patients (17.74%) had university degree or higher. In control group, 38 were males and 24 were females, with mean age of (51.96±13.19) years. As regards educational background, the corresponding number of cases was 42 (67.74%), 18 (29.03%) and 2 (3.23%). The average hospitalization time of patients was 21.5 days. Difference of gender, age, education background and clinical diagnosis of patients between two group were not statistically significant (P>0.05).

### Conventional nursing intervention

Control group received conventional nursing intervention. Charge nurse informed patients with cause of lung cancer, treatment method, treatment effect, prognosis,^7^ matters needing attention before and after surgery such as diet, rest, knowledge relating to management of pain on wound and various exercise methods. One day before surgery, operating room nurse informed patients with operating time, anesthesia method, preparation before surgery, pain and bleeding that may occur after surgery and matters needing attention after surgery.

### Comprehensive psychological intervention

First term responsible for intervention was set up. Related staffs were taught with intervention content and method as well as pain evaluation method. Patients in experimental group were given the following intervention measures,^8^,^9^ besides conventional nursing.^8^,^9^ First was psychological support from family. Staff inform family members of patients with related knowledge and importance of psychological intervention, suggested them to care and encourage patients before and after surgery continuously and coordinated medical staffs’ psychological intervention on patients. The second step was supportive psychological intervention from patients who also developed lung cancer. Five to six patients who developed lung cancer but recovered well after surgery were asked to gather and communicate with patients in experimental group to pour out feelings and share experience and importance of treatment compliance. The third step was supportive psychological intervention in aspect of language. Nurses and doctors should pay attention to their language expression and encourage and comfort them during treatment, thus to increase patients’ sense of security. The fourth step was imagery therapy for postoperative pain. Besides three step analgesic ladder, kinesitherapy and physical therapy, patients also received imagery therapy. Nurses guided patients to recall the happiest and most successful things in the life and image that surgery could completely remove tumor and body could recover after surgery.

### Detection of immune function

Fasting blood was collected from patients on the second day after being admitted into hospital and one day before leaving hospital. Two ml blood loaded in tube was sent for detecting content of T cell subgroups such as CD3^+^, CD4^+^, CD8^+^ as well as CD4^+^/CD8^+^ within 30 minutes. Meanwhile, 2 ml blood was put in anticoagulation tube and centrifuged at a speed of 3,000 r/minutes. The plasma isolated was then preserved at - 84 °C. Moreover, antibody was detected using CANTO type II flow cytometry (Becton, Dickinson and Company, USA) in the laboratory of hematology department in the hospital. Normal scope of CD3^+^, CD4^+^ and CD8^+^ is 50%~84%, 27%~51% and 15%~44% respectively. A centralized detection was performed on free cortisol. Free cortisol level was measured using Bayer Centaur immunity analyzer and its corresponding kit with enhanced chemiluminescence assay in the laboratory of endocrinology department in the hospital. Free cortisol with 118.6~618.0 nmol/L is considered to be normal.

### Evaluation of pain

Visual analog scale was used to evaluate pain; number 1 ~ 10 stands for different strength grades.^10^ Zero point refers to no pain, and 10 points refers to intolerable pain. Mild pain is scored for 1 ~ 3 points, medium pain is scored for 4 ~ 6 points and severe pain is scored for 7 ~ 9 points. VAS was recorded before anesthesia as well as 6 hour, 12 hour, 24 hour, and 48 hour after surgery.

### Statistical processing

All data were analyzed with SPSS 19.0 statistics software. Enumeration data were analyzed with chi-square test. Measurement data were expressed by mean ± SD. Comparison between groups was performed with one-way analysis of variance. Variance was converted before statistical analysis if it was not homogeneous. Difference was considered to have statistical significance if P<0.05.

## RESULTS

### Comparison of VAS at different time points

**Fig.1:**
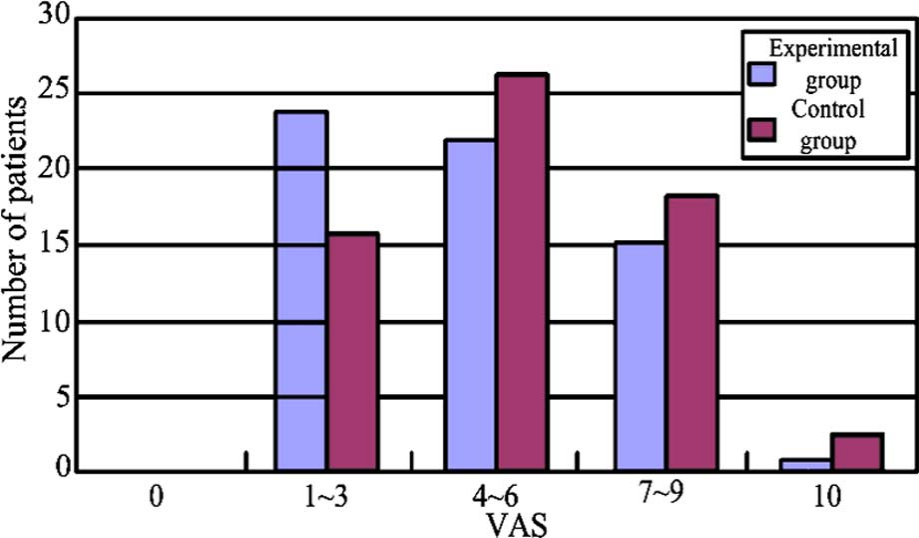
Number of cases with different grade of VAS 48 h after surgery.

Compared to control group, VAS of experimental group had a remarkable decrease before anesthesia, 6 h, 12 h, 24 h and 48 h after surgery (P<0.05). Data are shown in Table-I. 48 h after surgery, we found that, number of cases with high VAS was much less than control group. Numbers of cases with different grade of VAS 48 h after surgery are shown in Fig.1.

**Table-I:** Comparison of VAS between two groups at different time point (Mean ± SD).

Group	n	Before anesthesia	6 h after surgery	12 h after surgery	24 h after surgery	48 h after surgery
Control group	62	5.43±1.26	6.24±1.03	6.82±1.64	7.26±1.26	5.08±1.02
Experimental group	62	2.68±1.93*	3.22±1.84*	3.84±1.95*	4.24±1.43*	3.02±1.28*

* P < 0.05, compared to control group.

### Comparison of immune index and free cortisol before and after intervention

In experimental group, CD3^+^, CD4^+^ and CD4^+^/CD8^+^ increased after psychological intervention, but CD8^+^ declined; difference was not statistically significant before and after nursing within group, but difference between experimental group and control group was (P<0.05). In control group, CD3^+^, CD4^+^ and CD4^+^/CD8^+^ slightly rose after nursing, while CD8^+^ declined; difference was of no statistical significance before and after intervention. Content of free cortisol in serum decreased in both groups after intervention, but difference within group was not statistically significant (P>0.05), but difference between experimental group and control group was (P<0.05). Detailed data are presented in Table-II.

**Table-II:** Comparison of immune index and free cortisol before and after intervention in two groups (Mean ± SD).

Group	n		CD3^+^ (%)	CD4^+^(%)	CD8^+^(%)	CD4^+^/CD8^+^ (%)	Free cortisol (nmol/L)
Experimental group	62	Before intervention	64.19±9.42^a^	35.81±7.93^a^	27.17±8.89	1.48±0.69	576.67±125.11^a^
		After intervention	67.19±8.84	39.93±8.09^b^	25.95±8.12	1.70±0.73	507.97±131.90
Control group	62	Before intervention	65.35±11.26	39.22±7.61	24.30±8.60	1.87±0.99	494.68±153.58
		After intervention	69.28±11.12	42.06±10.00	25.29±8.15	1.83±0.70	471.26±91.67

a P<0.05 in comparison between experimental group and control group;

b P<0.05 in comparison before and after intervention in experimental group.

### QLQ-C30 scale score before and after intervention in two groups

Except physical items, difference of other items before and after intervention in experimental group had statistical significance and moreover, quality of life improved (P<0.05). But in control group, only symptoms improved, and other items had no meaningful changes; quality of life was also not enhanced (P>0.05). Except physical symptoms, difference of other items before and after intervention was statistically significant between groups (P<0.05). Data are presented in Table-III.

**Table-III:** Comparison of QLQ-C30 scale score between two groups (point, Mean ± SD)

Group	n		Social role	Body	Recognition	Emotion	Symptoms	Whole
Experimental group	62	Before intervention	7.01±2.18	7.85±2.02	2.76±1.02	5.41±1.79	16.35±3.08	7.85±2.52
		After intervention	8.19±2.10^a^^b^	8.47±2.47	3.29±1.21^a^^b^	7.91±2.24^a^^b^	18.76±3.78^a^^b^	10.65±2.02^a^^b^
Control group	62	Before intervention	6.76±2.39	14.00±2.22	2.98±0.91	7.24±2.31	17.57±3.51	8.75±2.38
		After intervention	6.87±2.24	8.25±2.74	3.24±0.98	7.81±1.95	19.59±4.43^c^	8.22±2.07

a P<0.05, in comparison of score before and after intervention between experimental group and control group;

b P<0.05, in comparison of score before and after intervention in experimental group;

c P<0.05, in comparison of score before and after intervention in control group.

## DISCUSSION

Lung cancer is one of the malignant tumors with high incidence and death rate severely threatens people’s life and health. It is reported that,^11^ lung cancer ranks the first place among malignant tumor in developed countries, and its incidence and death rate become increasingly higher in recent year, especially in city. Once lung cancer is confirmed, patients will be affected physically and mentally, and psychological stress may be induced in the process.^12^ What is worse, surgery as an effective means for treating lung cancer is also able to result in severe physical reactions,^13^ aggravating negative emotion of patients. Pain occurring after lung cancer, the most common and painful physical symptom, can also be induced under the influence of surgical trauma, constitution and psychological quality.^14^ Postoperative pains involving multiple factors are severe and complex, inducing not only tissue injury but also depression and anxiety. Therefore, psychological condition is considered to have large effect on curative effect; comprehensive psychological intervention is necessary.

It has been proved that,^15^ lung cancer patients who underwent surgery usually have psychological problem and proper psychological intervention can reduce negative emotion of patients and encourage them to face cancer positively. It can relieve pain induced by psychological factor. This study found that, VAS of experimental group was much lower than control group 6 hour, 12 hour, 24 hour and 48 hour after surgery (P < 0.05), indicating comprehensive nursing intervention could lower score and relieve pain after surgery.

Additionally, it has also been found that,^16^ psychological therapy can adjust patients’ emotion, relieve physical symptoms, enhance immune effect, improve quality of life, improve prognosis and prolong survival time. That is because, physical arousal level of sympathetic nerve is regulated under the influence of changed release of hypothalamic-pituitary-adrenal axis which is caused by reduced negative emotion, leading to changes of endorphin level and finally affecting immune index. The current study also suggested that, difference of immune index between experimental group and control group was statistically significant, indicating psychological intervention could improve immunoregulation capability of patients to some extent.

## CONCLUSION

To sum up, comprehensive psychological intervention can effectively relieve postoperative pain, improve immunoregulation capability, enhance psychological health and raise quality of life for lung cancer patients; therefore, it is worth further promotion and application.
